# Supplementary material for: COZOID: contact zone identifier for visual analysis of protein-protein interactions
Source: BMC Bioinformatics. 2018 Apr 6;19:125. doi: 10.1186/s12859-018-2113-6 (PMC5889581; doi:10.1186/s12859-018-2113-6)
Supplement: Supplementary file 2 — Software build. Executable binary file of the software tool. (ZIP 118,784 kb) [file 12859_2018_2113_MOESM2_ESM.zip › cozoid/java/docs/beansbinding-1.2.1-doc.zip!/org/jdesktop/swingbinding/class-use/JTableBinding.ColumnBinding.html]

Uses of Class org.jdesktop.swingbinding.JTableBinding.ColumnBinding (Beans Binding)


---


|  |  |  |
| --- | --- | --- |
| **Overview** \| **Package** \| **Class** \| **Use** \| **Tree** \| **Deprecated** \| **Index** \| **Help** | |  |
| PREV   NEXT | **FRAMES**    **NO FRAMES**     **All Classes** |  |


---


## **Uses of Class org.jdesktop.swingbinding.JTableBinding.ColumnBinding**

| Packages that use JTableBinding.ColumnBinding | |
| --- | --- |
| **org.jdesktop.swingbinding** | Provides support for binding to complex Swing components, and documentation on the interesting Swing properties to bind to. |

Uses of JTableBinding.ColumnBinding in org.jdesktop.swingbinding

| Methods in org.jdesktop.swingbinding that return JTableBinding.ColumnBinding | |
| --- | --- |
| `JTableBinding.ColumnBinding` | `JTableBinding.addColumnBinding(int index, Property<E,?> columnProperty)`             Creates a `ColumnBinding` and inserts it at the given index into the list of `ColumnBindings` maintained by this `JTableBinding`. |
| `JTableBinding.ColumnBinding` | `JTableBinding.addColumnBinding(int index, Property<E,?> columnProperty, java.lang.String name)`             Creates a `ColumnBinding` and inserts it at the given index into the list of `ColumnBindings` maintained by this `JTableBinding`. |
| `JTableBinding.ColumnBinding` | `JTableBinding.addColumnBinding(Property<E,?> columnProperty)`             Creates a `ColumnBinding` and adds it to the end of the list of `ColumnBindings` maintained by this `JTableBinding`. |
| `JTableBinding.ColumnBinding` | `JTableBinding.addColumnBinding(Property<E,?> columnProperty, java.lang.String name)`             Creates a named `ColumnBinding` and adds it to the end of the list of `ColumnBindings` maintained by this `JTableBinding`. |
| `JTableBinding.ColumnBinding` | `JTableBinding.getColumnBinding(int index)`             Returns the `ColumnBinding` with the given index in the list maintained by this `JTableBinding`. |
| `JTableBinding.ColumnBinding` | `JTableBinding.removeColumnBinding(int index)`             Removes the `ColumnBinding` with the given index from the list maintained by this `JTableBinding`. |
| `JTableBinding.ColumnBinding` | `JTableBinding.ColumnBinding.setColumnClass(java.lang.Class<?> columnClass)`             Sets the column class to be used by `JTable` to determine the renderer and editor for the column represented by this `ColumnBinding`. |
| `JTableBinding.ColumnBinding` | `JTableBinding.ColumnBinding.setColumnName(java.lang.String name)`             Sets a name for the column represented by this `ColumnBinding`. |
| `JTableBinding.ColumnBinding` | `JTableBinding.ColumnBinding.setEditable(boolean editable)`             Sets whether or not the cells of the column should be editable. |

| Methods in org.jdesktop.swingbinding that return types with arguments of type JTableBinding.ColumnBinding | |
| --- | --- |
| `java.util.List<JTableBinding.ColumnBinding>` | `JTableBinding.getColumnBindings()`             Returns an unmodifiable copy of the list of `ColumnBindings` maintained by this `JTableBinding`. |

| Methods in org.jdesktop.swingbinding with parameters of type JTableBinding.ColumnBinding | |
| --- | --- |
| `boolean` | `JTableBinding.removeColumnBinding(JTableBinding.ColumnBinding binding)`             Removes the given `ColumnBinding` from the list maintained by this `JTableBinding`. |

---


|  |  |  |
| --- | --- | --- |
| **Overview** \| **Package** \| **Class** \| **Use** \| **Tree** \| **Deprecated** \| **Index** \| **Help** | |  |
| PREV   NEXT | **FRAMES**    **NO FRAMES**     **All Classes** |  |


---
